# Adenoviruses Encapsulated in PEGylated DOTAP-Folate Liposomes Are Protected from the Pre-Existing Humoral Immune Response

**DOI:** 10.3390/pharmaceutics17060769

**Published:** 2025-06-11

**Authors:** Abraham T. Phung, Jaimin R. Shah, Tao Dong, Omonigho Aisagbonhi, William C. Trogler, Andrew C. Kummel, Sarah L. Blair

**Affiliations:** 1 Moores Cancer Center, University of California San Diego, San Diego, CA 92037, USA; a1phung@ucsd.edu (A.T.P.); j2shah@ucsd.edu (J.R.S.); t2dong@ucsd.edu (T.D.); oaisagbonhi@health.ucsd.edu (O.A.); 2 Department of Chemistry and Biochemistry, University of California San Diego, San Diego, CA 92037, USA; wtrogler@ucsd.edu (W.C.T.); akummel@ucsd.edu (A.C.K.); 3 Department of NanoEngineering, University of California San Diego, San Diego, CA 92037, USA; 4 Program in Materials Science and Engineering, University of California San Diego, San Diego, CA 92037, USA; 5 Department of Pathology, University of California San Diego, San Diego, CA 92037, USA; 6 Department of Surgery, University of California San Diego, San Diego, CA 92037, USA

**Keywords:** liposome, adenovirus, PEG, neutralizing antibodies, systemic administration

## Abstract

**Background/Objectives**: While adenovirus (Ad) therapies have been proven to be effective in local administration, systemic Ad treatments have shown limited success due to pre-existing antibodies in the human blood that neutralize the virus. We developed a liposome coating procedure that protects the Ad from pre-existing neutralizing antibodies in human blood. To assess the in vivo stability of the liposomes, the present study used a novel in vivo method to quantitatively assess the protective capabilities of liposome-encapsulated Ad (DfAd) from neutralizing antibodies. **Methods**: The assay systemically administers DfAd with a green fluorescent protein transgene (DfAd-GFP) into pre-immunized mice and allows it to circulate in the presence of neutralizing antibodies; the infected blood is extracted and used to transduce HEK293 cells, which emits fluorescence in the presence of protected, un-neutralized Ad. **Results**: The PEGylated liposome formulation provides 12× protection in vivo relative to unencapsulated Ads. In vitro optimization of the liposome coating reveals a strong correlation between the structural stability of liposomes and protection against anti-Ad neutralizing antibodies, where DSPE-PEG2000-carboxylic acid (DSPE-PEG2000-CA) is a critical component for liposome stability and increasing protection against antibody neutralization of the encapsulated Ad. **Conclusions**: The findings in the present study confirm that the DfAd liposome can protect against neutralizing antibodies in blood circulation. The novel in vivo assay for liposome protection against neutralizing antibodies and in vitro experiments in the present study provide new tools and insights toward designing liposome–Ad complexes for the systemic treatment of cancer.

## 1. Introduction

Oncolytic adenovirus (Ad) therapy is an emerging treatment for various cancers, including, but not limited to, breast [1,2,3], gastrointestinal [4], ovarian [5], and brain cancers [6]. The treatment involves infecting and killing cancer cells with an Ad engineered to conditionally replicate in cancer cells, thereby inducing cell death but remaining harmless to healthy cells. When administered intratumorally, oncolytic adenoviral therapy effectively trains the immune system to identify and kill disseminating tumor cells and metastatic tumors [7]. The anti-tumor immune response can also be amplified by engineering the oncolytic Ad genome with a highly expressible transgene, such as immune stimulatory peptides [8], or combining intratumorally administered oncolytic Ad treatment with immune-checkpoint inhibitors (ICI) [9]. While these approaches theoretically treat both the primary and distant lesions, recent studies show tumors that regress from direct treatment with oncolytic viruses respond better to treatment than distant tumors that only regress from anti-tumor immunity [10,11]. Although studies of intratumoral delivery of oncolytic Ad have successfully regressed not only the injected primary tumor but also distant, noninjected tumors, intravenous administration is still the ideal route of treatment for hard to reach tumors and multiple tumors at the same time [12]. In summary, systemic delivery of oncolytic viruses may be the best approach to controlling local tumor lesions and metastatic disease compared with intratumoral delivery [13].

Current endeavors to systemically administer oncolytic Ad face many challenges, including dilution, opsonization, and neutralization [10,14,15]. Intravenous administration of oncolytic Ad significantly dilutes the administered dose. In conjunction with pre-existing humoral immunity against Ads in humans and liver macrophages that actively sequester foreign viral particles, the efficacy of systemically administered oncolytic Ad is greatly reduced by the lack of viable virions reaching the target tumor site. Previous dose escalation studies showed that high-dose intravenous administration of oncolytic Ad is well tolerated, with no dose-limiting toxicities observed; however, in a few cases, flu-like symptoms have been observed [16,17,18]. Despite high-dose intravenous administration, these studies still showed low uptake of viral particles, with most of the virus being neutralized or sequestered by the liver [16,17,18,19,20,21]. Therefore, solutions to prevent adenoviral neutralization are critical for the clinical translation of systemically administered oncolytic viral therapy. One approach is to protect the Ad by encapsulation in a liposome.

**Figure 1:**
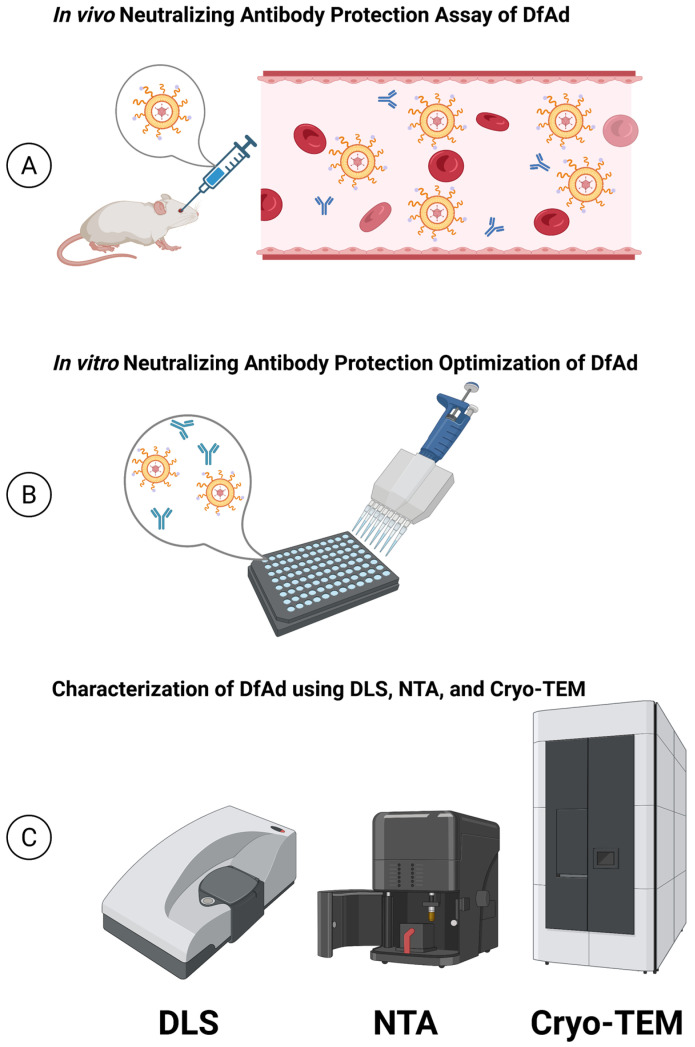
Experimental framework for the present study. The present study is organized around three main experiments. (**A**) The in vivo neutralizing antibody protection assay was performed on immunized mice to evaluate the in vivo neutralizing antibody protection capabilities of DfAd. (**B**) The in vitro neutralizing antibody protection assays were performed to optimize the DfAd formulation. (**C**) DfAd was characterized using dynamic light scattering (DLS), nanoparticle tracking analysis (NTA), and cryogenic transmission electron microscopy (cryo-TEM). Created in BioRender. Phung, A. (2025) https://BioRender.com/31uduc2. Accessed on 26 May 2025.

Liposome-encapsulated Ad has been investigated for improving transduction in various tumors, especially tumors with negative coxsackievirus and Ad receptor (CAR) expression [22,23,24,25,26]. While previous studies have shown the ability of liposomes to protect Ad from neutralizing antibodies in vitro [27,28], there is little evidence of liposomes protecting Ad in vivo during systemic administration, and few published methods quantitatively assess in vivo protection capabilities of liposomes [29]. Liu et al. is one of the few published studies that explicitly describe an in vivo assay to measure in vivo neutralizing antibody protection of liposome-encapsulated Ad in pre-immunized mouse models [29]. However, this assay relies on liver uptake of the virus and a protocol that measures in vivo antibody neutralization of naked and liposome-protected oncolytic viruses is needed. Inspired by the method described in Liu et al. and blood viral titer measurements, the present study describes a novel method of measuring neutralizing antibody protection. This involves measuring the transduction efficiency of serum isolated from pre-immunized, systemically infected mice. Using the experimental framework in Figure 1, we demonstrate the in vivo protection capabilities of PEGylated DOTAP-folate liposome-encapsulated Ad (DfAd) and describe the critical components that allow DfAd to protect against neutralizing antibodies.

## 2. Materials and Methods

### 2.1. Reagents and Cell Lines

Replication-deficient Ad expressing green fluorescent protein (Ad-GFP) was purchased from Baylor College of Medicine, Houston, TX, USA (Catalog: Ad5-CMV-eGFP). The HEK293 cell line was purchased from ATCC, Manassas, VA, USA (Catalog: CRL-1573). HEK293 was cultured in complete media of Dulbecco’s Modified Eagle Medium (DMEM) with high glucose (Fisher Scientific, Hampton, NH, USA #SH3024301) supplemented with 10% FBS (Omega Scientific, Tarzana, Los Angeles, CA, USA #FB-02) and 1% penicillin–streptomycin–glutamin (PSG, Life Technologies, Carlsbad, CA, USA #10378016).

### 2.2. Synthesis of Liposome-Encapsulated Ad-GFP

Liposome encapsulation of Ad was performed by extrusion, which has been described previously [24]. In brief, DOTAP (Avanti, Alabaster, AL, USA #890890C), cholesterol (Sigma, Saint Louis, MO, USA #C3045), 1,2-distearoyl-*sn*-glycero-3-phosphoethanolamine-N-[carboxy(polyethylene glycol)-2000] [PEG(2000)-PE carboxylic acid, Avanti #880124P], and 1,2-distearoyl-*sn*-glycero-3-phosphoethanolamine-N-[folate(polyethylene glycol)-2000] [PEG(2000)-Folate-PE, Avanti #880124P] were mixed together in chloroform at a molar ratio of 1:0.26:0.02:0.01. To make 400 µL of DOTAP-folate Ad-GFP (DfAd-GFP), 387 nmol of DOTAP, 100 nmol of cholesterol, 7.01 nmol of PEG(2000)-PE carboxylic acid, and 3 nmol of PEG(2000)-folate-PE was added to 193.13 µL of chloroform (Sigma, Saint Louis, MO, USA C2432) in an amber vial (Fisher Scientific #03-339-23C). The lipid mixture was vortexed for 30 min at 25 °C. The resulting mixture was vacuumed overnight to form a dry lipid film at the bottom of the vial. The next day, the dry film was rehydrated with 400 µL of phosphate buffered saline (PBS, Fisher Scientific, Hampton, NH, USA #10010072) while vortexing. The hydrated film was stirred at 600 rpm for 30 min at 4 °C. Empty liposomes were formed by extruding the lipid mixture with the Avanti Mini Extruder (Avanti, Alabaster, AL, USA #6100009-1EA) through a 200 nm membrane (Cytiva/Whatman, UK #10417004), 8 times at room temperature. To the empty liposomes, Ad-GFP was added to reach 5 × 10^10^ viral particles (vp) mL^−1^, and the mixture was incubated at room temperature for 30 min to allow for encapsulation of the Ad-GFP. The resulting extruded DfAd-GFP has an Ad to DOTAP lipid ratio [viral particles (VP): nmol] of 5.17 × 10^7^ [24].

### 2.3. In Vivo Neutralizing Antibody Protection Assay

Six-to-eight-week-old female BALB/c mice were purchased from the Jackson laboratory, Bar Harbor, ME, USA. Mice were housed in high-efficiency particulate air (HEPA) cages in a specific pathogen-free facility with food and water available and a 12 h light/dark cycle. Six mice were immunized with an injected in the right flank with 5 × 10^8^ viral particles (vp) of unencapsulated Ad (wild-type Ad, Vector Biolabs, Malvern, PA, USA) and another six with an equivalent volume of PBS. Afterwards, the immunized mice developed neutralizing antibodies for at least 21 days. Pain and distress in immunized mice were closely monitored. After a minimum of 21 days, six mice (three immunized with Ad and three immunized with PBS) were retro-orbitally (RO) injected with 5 × 10^9^ vp of DfAd-GFP and the other six with Ad-GFP under isoflurane sedation. The injected orbital sinus was immediately anesthetized with 0.5% proparacaine hydrochloride ophthalmic drops (Sigma Pharmaceuticals, North Liberty, IA, USA #4503-2). Pain and distress in the RO-injected mice were closely monitored. After waiting 5 min, mice were sacrificed, and blood was collected from each mouse using cardiac puncture. All procedures and protocols were approved by the UC San Diego Institutional Animal Care and Use Committee (IACUC).

Serum was isolated from each mouse blood sample by leaving the blood to coagulate in an Eppendorf tube on ice for 30 min. Each blood sample was centrifuged at 400× *g* for 5 min at 4 °C, and the top layer of serum was collected. HEK293 cells plated at 3 × 10^4^ cells per well in 96-well plates were then transduced with 50 µL of each serum sample (day 1). The cells were then incubated at 37 °C at 5% CO_2_ for 24 h. GFP fluorescence intensities were measured using a Tecan F PLEX Infinite 200 Pro microplate reader on day 2.

### 2.4. Fluorescence Microscopy

Cells transduced with DfAd-GFP or Ad-GFP were analyzed under a Keyance BZ-X710 microscope (KEYENCE CORPORATION OF AMERICA, Itasca, IL, USA) with a GFP filter and 470/40 nm excitation wavelength, 525/50 nm emission wavelength and dichroic mirror wavelength of 495 nm. Comparative micrographs were captured using 2× and 20× objective lenses. Cell counting was performed using images taken with the 20× objective lens.

### 2.5. In Vitro Neutralizing Antibody Protection Assay

HEK293 cells were plated at between 1 × 10^4^ to 3 × 10^4^ cells well^−1^ in 96-well plates and incubated at 37 °C and 5% CO_2_ in complete media. A neutralizing antibody protection assay was performed after cells were attached to the well (24–48 h after plating). Media in the wells was removed from the wells without disturbing the cells. DfAd-GFP or Ad-GFP was mixed with 1/10× serum (human or mouse) and added to the cells at MOI 100 with enough volume to cover the cells (day 1). Cells were then incubated at 37 °C and 5% CO_2_ for 1 h before the mixture was removed from the wells and replaced with fresh complete media, trying not to disturb the cells. The cells were incubated again at 37 °C and 5% CO_2_ for 24 h. GFP fluorescence intensities were measured using a Tecan F PLEX Infinite 200 Pro microplate reader (Tecan Group Ltd., Männedorf, Switzerland) on day 2.

### 2.6. Cryo-Transmission Electron Microscopy (Cryo-TEM)

Quantifoil carbon R2/2 copper grids were acquired from Quantifoil Micro Tools GmbH (Jena, Germany). Cryo-TEM imaging was performed using a JEOL JEM-2100F transmission microscope (JEOL USA, Peabody, MA, USA). Before imaging, grids were glow discharged using a Solarus Plasma Cleaner 950 (Gatan, Inc., Pleasanton, CA, USA). An amount of 3 µL of each sample was applied to a grid before sample freezing. Blotting and freezing were performed at room temperature and 95% humidity using a Leica EMGP plunger (Leica Microsystems Inc., Deerfield, IL, USA). Blotting time was set to 3 s without waiting or draining steps. Frozen grids were loaded on a cold Gatan cryo-transfer holder (Gatan, Inc., Pleasanton, CA, USA) and imaged at 30,000× magnification. Images were captured on a Gatan OneView CCD (Gatan, Inc., Pleasanton, CA, USA) with SerialEM software 4.2.0 (University of Colorado, Boulder, CO, USA). Images were processed using Fiji 2.16.0/1.54p.

### 2.7. Statistical Analysis

Prism 10.4.1 software (GraphPad Software LLC, Boston, MA, USA) was used for data analysis. Comparison between the two groups was based on a two-tailed unpaired *t*-test. A value of *p* < 0.05 was determined to be statistically significant.

## 3. Results

### 3.1. In Vivo Neutralizing Antibody Protection Assay

**Figure 2:**
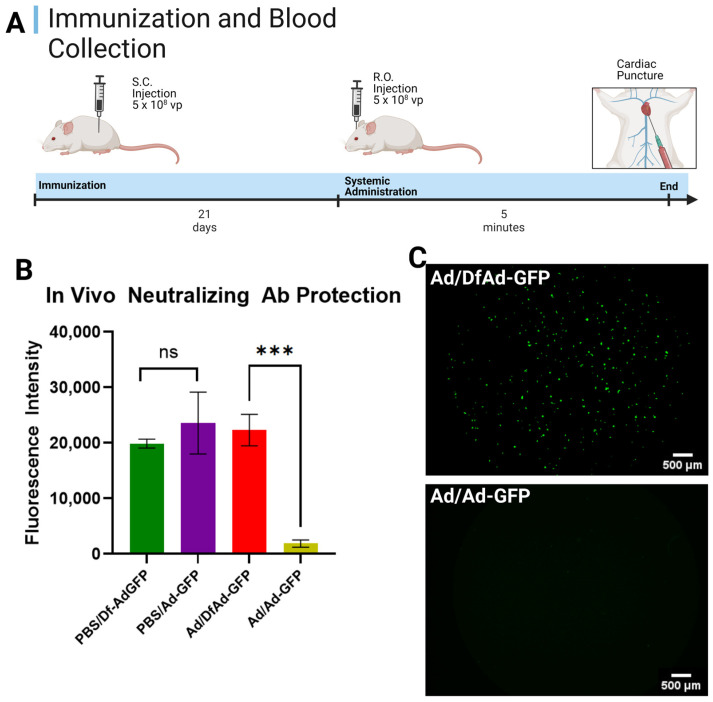
In vivo neutralizing antibody protection of DfA against pre-existing anti-Ad antibodies. (**A**) Schedule for mouse immunization, sample administration, and cardiac puncture. Immunocompetent Balb/c mice are immunized subcutaneously with 5 × 10^8^ vp of Ad or an equal volume of PBS. Twenty-one days later, mice are RO-treated with 5 × 10^8^ vp DfAd-GFP or Ad-GFP; 5 min later, mice are sacrificed, and blood is collected via cardiac puncture. (**B**) In vivo neutralizing antibody protection assay performed by transducing HEK293 cells with serum from PBS or Ad-immunized mice systemically treated with either Df-AdGFP or Ad-GFP. PBS/DfAd-GFP = transduction efficiency of serum extracted from mice immunized with PBS and systemically injected with DfAd-GFP (green). PBS/Ad-GFP = transduction efficiency of serum extracted from mice immunized with PBS and systemically injected with Ad-GFP (purple). Ad/DfAd-GFP = transduction efficiency of serum extracted from mice immunized with Ad and systemically injected with DfAd-GFP (red). Ad/Ad-GFP = transduction efficiency of serum extracted from mice immunized with Ad and systemically injected with Ad-GFP (gold). *** *p* < 0.001, *n* = 4. (**C**) Fluorescent micrographs of HEK293 cells transduced with serum from a mouse immunized with Ad and systemically injected with Df-AdGFP (**top**) and a mouse immunized with Ad and systemically injected with Ad-GFP (**bottom**). Both images were auto leveled using the preview application to improve contrast. Created in BioRender. Phung, A. (2025) https://BioRender.com/4eh0ucc. Accessed on 26 May 2025.

Figure 2A shows the mouse immunization and injection schedule for the in vivo neutralizing antibody protection assay, which will measure the protection by the liposomal coating against neutralizing anti-Ad antibodies in circulation inside of immunized mice. After mice were immunized with Ad for 21 days, they were systemically administered DfAd-GFP or Ad-GFP, which were allowed to circulate for 5 min before the mice were sacrificed and their blood was collected. Five minutes of circulation time equates to at least 20 passes through the mouse circulatory system and provides sufficient exposure time to neutralizing antibodies with minimal involvement of liver and macrophage clearance systems [30,31]. The number of viable viruses in the blood was assessed by transfecting HEK293 cells with isolated serum infected with DfAd-GFP or Ad-GFP and fluorescence was read using a Tecan fluorescent plate reader.

Figure 2B shows the results of the in vivo neutralizing antibody assay on mice immunized by PBS and systemically injected with DfAd-GFP (the green bar, PBS/Df-Ad-GFP); immunized by PBS and systemically injected with Ad-GFP (the purple bar, PBS/Ad-GFP); immunized by Ad and systemically injected with DfAd-GFP (the red bar, Ad/DF-Ad-GFP); and immunized by Ad and systemically injected with Ad-GFP (gold bar, Ad/Ad-GFP). The assay shows that the serum of mice that were immunized with PBS showed no difference in transduction efficiency in HEK293 cells, whether they were systemically injected with DfAd (green) or Ad (purple). This result confirms that mice were consistently injected with the same number of viral particles and that other Ad clearance systems were minimally involved. However, when mice were immunized with Ad, serum from mice that were systemically injected with DfAd-GFP (red, Ad/DF-Ad-GFP) demonstrated 12× higher transduction efficiency compared with serum of mice that were injected with unencapsulated Ad-GFP (gold, Ad/Ad-GFP). This result shows that DfAd-GFP was protected from circulating neutralizing antibodies in immunized mice and that more viable DfAd-GFP were recovered in serum compared with unencapsulated Ad-GFP. Significantly fewer Ad-GFP were recovered in serum without liposome protection in vivo, resulting in 12× lower transduction efficiency. Figure 2C shows fluorescent micrographs of wells containing HEK293 cells that were treated with serum from a mouse that was Ad-immunized and RO-injected with DfAd-GFP (top image) and cells that were treated with serum from a mouse that was Ad-immunized and RO-injected with unencapsulated Ad-GFP. The top image demonstrates significantly more fluorescent HEK293 compared with the bottom image, which shows no fluorescent HEK293 cells.

### 3.2. In Vitro Optimization of DfAd for Protection Against Neutralizing Antibodies

**Figure 3:**
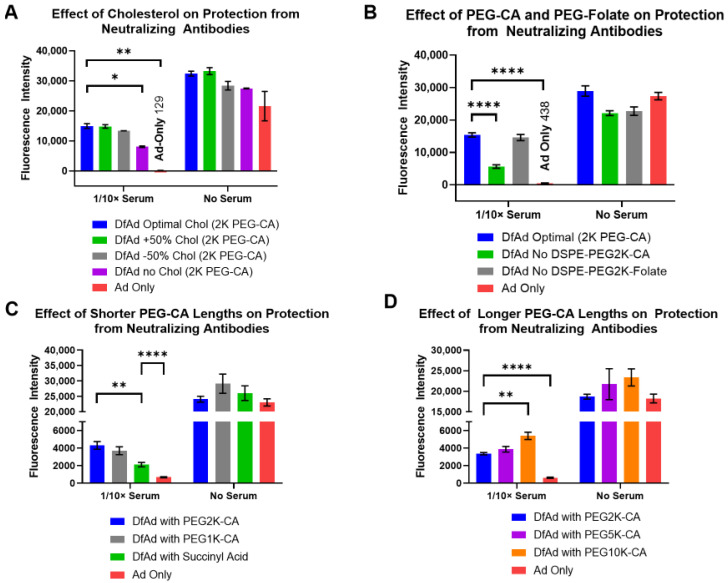
Effect of liposome formulation on in vitro protection from neutralizing antibodies. (**A**) The effect of cholesterol composition on liposomal protection from neutralizing antibodies. This experiment used neutralizing serum from immunized mice. * *p* ≤ 0.05, ** *p* ≤ 0.01, *n* = 2. (**B**) The effect of incorporating PEG2000-CA and PEG2000-Folate on liposomal protection from neutralizing antibodies. This experiment used neutralizing serum from immunized mice. **** *p* ≤ 0.0001, *n* = 4. (**C**) The effect of incorporating shorter PEG lipids on liposomal protection from neutralizing antibodies. This experiment used neutralizing serum from human blood samples. ** *p* ≤ 0.01, **** *p* ≤ 0.0001, *n* = 6. (**D**) The effect of incorporating longer PEG lipids on liposomal protection from neutralizing antibodies. This experiment used neutralizing serum from human blood samples. ** *p* ≤ 0.01, **** *p* ≤ 0.0001, *n* = 6.

The formulation of DfAd, cholesterol concentration, PEG lipid concentration, folate PEG lipid concentration, and PEG lipid length were adjusted to determine the critical components that provide DfAd protection against neutralizing antibodies in vitro. Experiments in Figure 3A,B used 10× diluted neutralizing serum from immunized mice so that there would be repeatable results between serum batches. Figure 3A shows the results of optimizing the cholesterol concentration in DfAd, where changing the concentration of cholesterol by 50% did not affect the protection against neutralizing antibodies, as evidenced by the marginal change in transduction efficiency in 1/10× mouse serum. Altogether omitting cholesterol from the DfAd formulation lowered the protection by ~50%. In Figure 3B, results show that removing DSPE-PEG2000-folate, the cancer-targeting lipid PEG peptide, from the DfAd formulation did not affect the protection against neutralizing antibodies. However, removing DSPE-PEG2000-CA (typically used to stabilize liposomes in aqueous solution) reduced protection by ~70%.

Experiments shown in Figure 3C,D used neutralizing serum from human blood samples to benchmark the protective capabilities of DfAd in serum with clinically relevant neutralizing antibody titers. The results show that replacing the DSPE-PEG2000-CA in the DfAd formulation with DSPE-PEG5000-CA or with DSPE-PEG1000-CA barely changed the protection against neutralizing antibodies. However, by replacing DSPE-PEG2000-CA with DSPE-succinyl acid (DSPE-CA, no PEG chain) or DSPE-PEG10k-CA, protection against neutralizing antibodies was lowered by ~50% or increased by about ~60%, respectively. The latter results suggest that extremely long PEG enhances protection against neutralizing antibodies.

### 3.3. Characterization of DfAd

Physical characterization was performed using the optimized formulation with single variations to determine the correlation between in-vitro neutralizing antibody protection and physical stability. Dynamic light scattering (DLS), nanoparticle tracking analysis (NTA), and transmission electron microscopy (TEM) were used to measure the size and assess the structural stability of DfAd.

**Figure 4:**
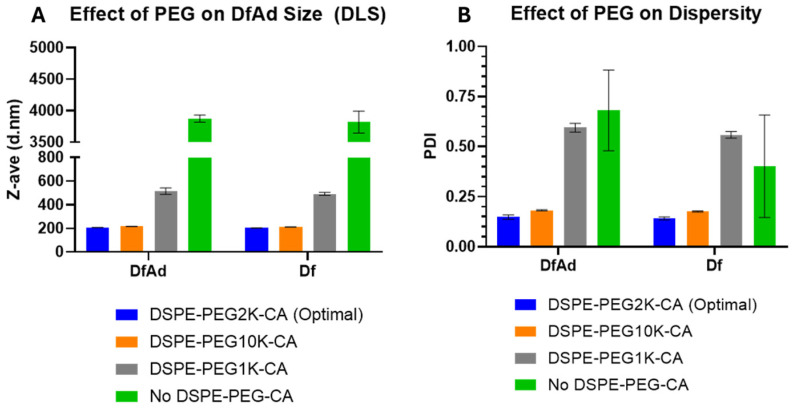
Characterization of various DfAd formulations using DLS. (Table 1) The effect of PEG lipid composition and length on the particle size using DLS on DfAd. Fold change refers to the relative change in z-average diameter or PDI of the DfAd sample of interest compared with the z-average diameter or PDI of DfAd with DSPE-PEG2000-CA. (**A**) Graphical representation of the effect of PEG on DfAd size. (**B**) Graphical representation of the effect of PEG on particle dispersity (uniformity).

As shown in Table 1 and Figure 4, DLS size measurements showed that the optimal DfAd with DSPE-PEG2K-CA was ~206 nm, with a uniform polydispersity index of 0.15. Note that all measurements in Table 1 are taken without stirring. Replacing the DSPE-PEG2000-CA with DSPE-PEG10K-CA in the DfAd formulation increased the DfAd hydrodynamic size to ~215 nm, while maintaining a uniform polydispersity of 0.18. However, by replacing DSPE-PEG2000-CA with DSPE-PEG1000-CA, the size of DfAd increased very slightly to ~514 nm, with an increased polydispersity index of 0.59. Completely removing DSPE-PEG2000-CA further increases the size of DfAd to ~3870 nm with an increased polydispersity index of 0.68. Figure 3A,B show graphical descriptions of the data in Table 1. They visually demonstrate the changes in size and dispersity between the DfAd with DSPE-PEG2K-CA and no DSPE-PEG-CA, where size increases by 19 fold, and dispersity increases 5 fold. The increased size and polydispersity with the removal of DSPE-PEG are consistent with the loss of physical stability of the DfAd, resulting in aggregation or flocculation of the nanoparticles. DfAd sizes were again measured using nanoparticle tracking analysis (NTA) to confirm these observations.

**Figure 5:**
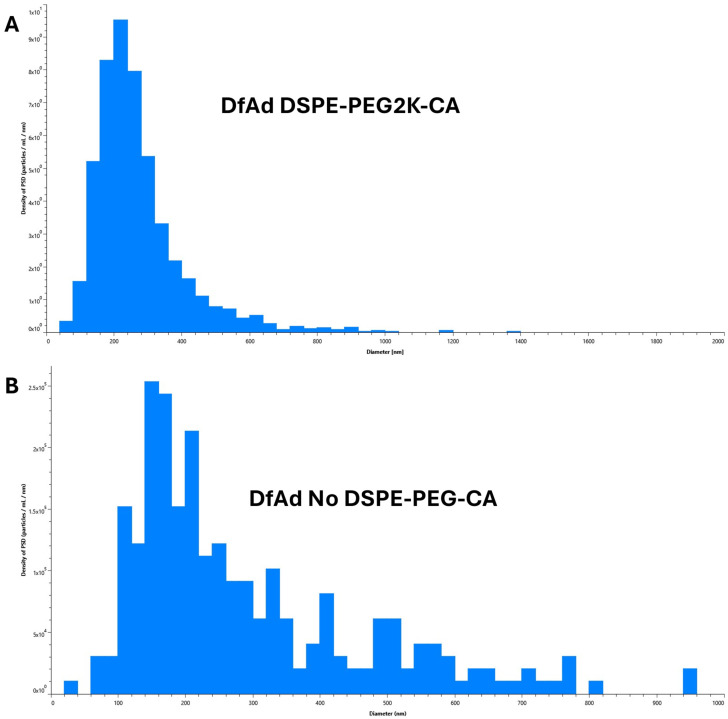
Characterization of various DfAd formulations using NTA without stirring. (Table 2) The effect of PEG lipid composition and length on particle size using NTA on DfAd without stirring. Fold change refers to the relative change in mean diameter of the DfAd sample of interest compared with the average diameter of DfAd with DSPE-PEG2000-CA. (**A**) Graphical size distribution of DfAd particles formulated with DSPE-PEG2K-CA. (**B**) Graphical size distribution of DfAd particles formulated without DSPE-PEG-CA.

NTA measurements in Table 2 and Figure 5 were taken of each sample without a stirring step between each read. With the removal of the stirring in between each read, the DfAd size was 136 nm. However, the DfAd without DSPE-PEG2000-CA increased by more than 2×, reaching 291 nm in diameter. Figure 5A,B show the particle size distributions of DfAd using DSPE-PEG2K-CA and no DSPE-PEG-CA, respectively, with a uniform distribution in Figure 5A and a more polydisperse distribution in Figure 5B. Increasing nanoparticle size with the removal of DSPE-PEG is consistent with flocculation, similar to the DLS experiment in Figure 4.

**Figure 6:**
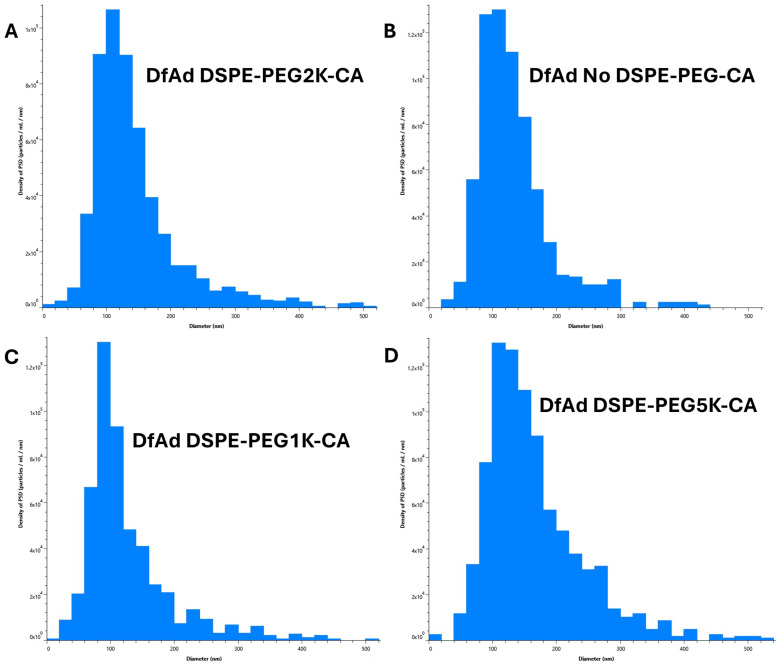
Characterization of various DfAd formulations using NTA with stirring. (Table 3) The effect of PEG lipid composition and length on particle size using NTA on DfAd with stirring. Fold change refers to the relative change in mean diameter of the DfAd sample of interest compared with the average diameter of DfAd with DSPE-PEG2000-CA. (**A**) Graphical size distribution of DfAd particles formulated with DSPE-PEG2K-CA. (**B**) Graphical size distribution of DfAd particles formulated without DSPE-PEG-CA. (**C**) Graphical size distribution of DfAd particles formulated with DSPE-PEG1K-CA. (**D**) Graphical size distribution of DfAd particles formulated with DSPE-PEG5K-CA.

NTA measurements shown in Table 3 and Figure 6 were first performed by stirring at 1400 RPM for 3 s between each read. NTA measurements showed that DfAd had a mean size of 145 nm. Replacing the DSPE-PEG2000-CA with DSPE-PEG1000-CA changed the size slightly to 128 nm, and replacing it with DSPE-PEG5000-CA changed the size slightly to 165 nm. Removing the DSPE-PEG2000-CA changed the size slightly to 134 nm. All four mean diameters are generally very similar, where all sizes are within ±10% of the DfAd DSPE-PEG2000-CA mean diameter. Figure 6A–D show the particle size distributions of DfAd using DSPE-PEG2K-CA, no DSPE-PEG-CA, DSPE-PEG1K-CA, and DSPE-PEG5K-CA, respectively, demonstrating uniform size distributions in all four samples. Figure 6A,B demonstrate very similar distributions, with their peaks hovering over ~120 nm. In Figure 6C, the curve is shifted very slightly to the left with the peak hovering over ~100 nm, and in Figure 6D is shifted very slightly to the right with the peak hovering over ~140 nm. Stirring between NTA reads likely breaks up flocculated nanoparticles and reestablishes uniformity in DfAd nanoparticle sizes, especially for the DfAd without DSPE-PEG-CA. The DfAd with DSPE-PEG2000-CA remains the same size with and without stirring, likely due to PEG preventing flocculation of the particles. NTA experiments demonstrate consistent observations made during DLS experiments, where DSPE-PEG2000-CA helps form smaller, more uniform nanoparticles while the removal of DSPE-PEG2000-CA forms larger, more polydisperse ones.

**Figure 7:**
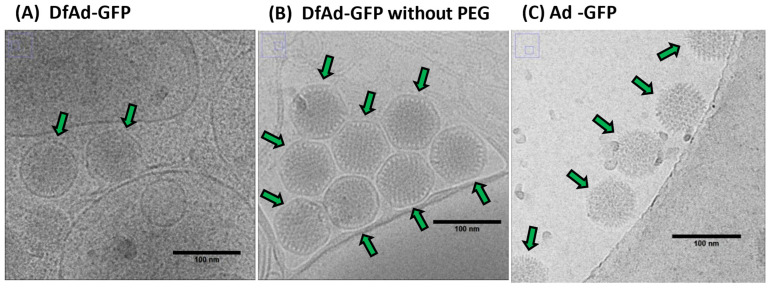
Characterization and structural analysis of DfAd. (**A**) Cryo-TEM image of DfAd-GFP at 30,000× magnification plus a 50% digital zoom. Green arrows point to liposome-encapsulated Ad-GFP. (**B**) Cryo-TEM image of DfAd-GFP without PEG at 30,000× magnification plus a 50% digital zoom. Green arrows point to liposome-encapsulated Ad-GFP without PEG. (**C**) Cryo-TEM image of Ad-GFP without encapsulation at 30,000× magnification plus a 50% digital zoom. Green arrows point to naked, unencapsulated Ad-GFP. In all figures, the scale bar for cryo-TEM images is 100 nm.

The cryo-TEM images in Figure 7 show that the Ad and the liposome coating can be distinguished. In Figure 7A,B, the liposome encapsulation appears to be of thin dark layers around darkened spheres of 70–80 nm in diameter. The diameter of the encapsulated dark spheres is around 80–90 nm. Figure 7A shows two separated DfAd nanoparticles marked with green arrows. Figure 7B shows eight DfAd nanoparticles bunched together, each marked with a green arrow. Liposome-encapsulated adenovirus in both Figure 7A,B are tightly enveloped with about 5–10 nm of space separating the surface of the virus from the lipid bilayer. While removing DSPE-PEG2000-CA did not change the size of the encapsulated virus, the DfAd without DSPE-PEG-CA appeared more bunched together, and the liposomes appeared to be more hexagonal than spherical, compared with the DfAd with DSPE-PEG2000-CA. In Figure 7C, the viruses are shown to be darkened spheres of roughly 70–80 nm in diameter. Five viral particles are each marked with green arrows. In Supplementary Figure S4A, there are two DfAd that are encapsulated together in one larger liposome. Supplementary Figure S4B shows DfAd without DSPE-PEG-CA, with similar shaped liposomes as in Figure 7B, except that the nanoparticles in Supplementary Figure S4B are less bunched together.

## 4. Discussion

**Figure 8:**
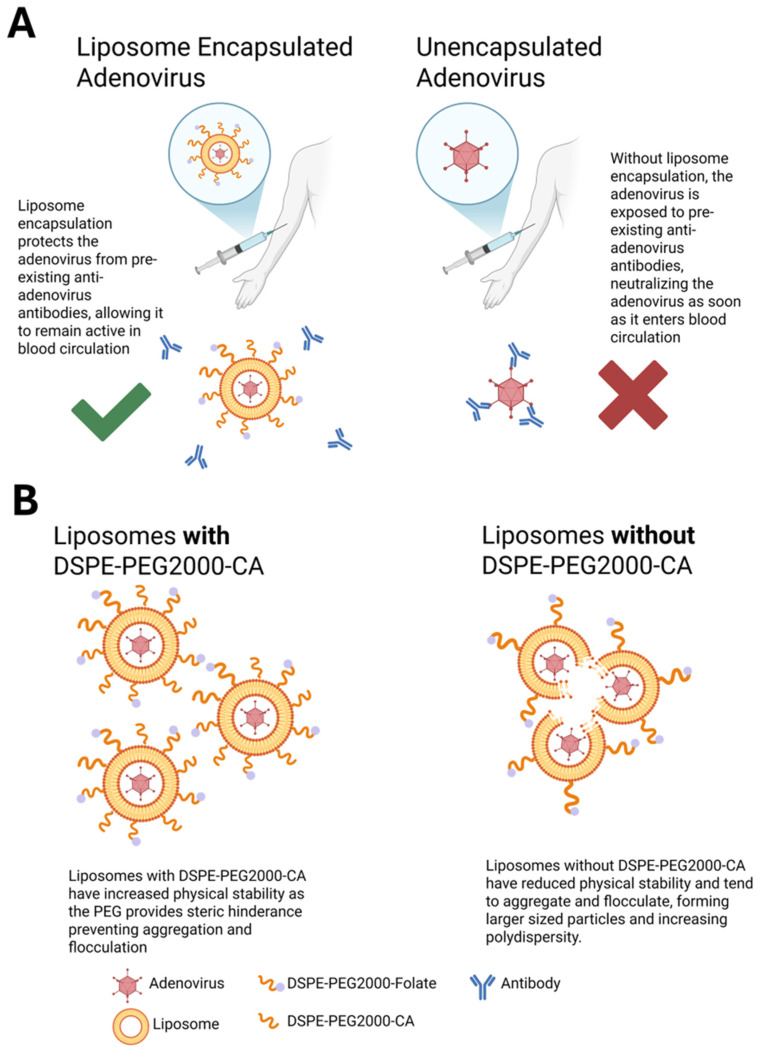
Summary schematic of PEGylated liposome-encapsulated Ad. (**A**) Schematic of liposome protecting encapsulated Ad from neutralizing antibodies in circulation. Without encapsulation, Ad is exposed to surrounding neutralizing antibodies in the blood and is immediately neutralized. (**B**) Schematic of DSPE-PEG2000-CA’s role in increasing stability of DfAd, resulting in smaller, more uniform nanoparticles. Removal of DSPE-PEG2000-CA results in reduced stability, causing aggregation between nanoparticles and increasing their size. (**A**) was created in BioRender. Phung, A. (2025) https://BioRender.com/hs8e533. Accessed on 26 May 2025. (**B**) was also created in BioRender. Phung, A. (2025) https://BioRender.com/j53c141. Accessed on 26 May 2025.

The present study demonstrates the in vivo protection of DfAd against neutralizing antibodies by directly exposing DfAd as well as unencapsulated Ad to circulating neutralizing antibodies and evaluating the viable virion after five minutes of circulation. Most of the DfAd remained un-neutralized during five minutes of circulation time in pre-immunized mice, while unencapsulated Ad were almost completely neutralized during the same circulation time. A five-minute circulation time equates to roughly 20 complete passes through a mouse’s blood system, assuming one pass is 15 s [31]. Figure 8A summarizes how DfAd is protected from pre-existing neutralizing antibodies, allowing for prolonged circulation while retaining most of its activity when compared with unencapsulated Ad, which becomes neutralized immediately when exposed to pre-existing antibodies and loses almost all activity. Previous studies encapsulating Ad with liposomes have also shown in vivo neutralizing antibody protection with similar platforms [29]. However, these studies evaluated in vivo protection by measuring liver cell transduction efficiency in immunized mouse models. While liver transduction could be an accurate method of evaluating in vivo neutralizing antibody protection, a more direct method is needed. The liver transduction method theoretically suggests that increased transduction of liver cells by encapsulated Ad in pre-immunized mice translates to increased protection. Because liver uptake of therapeutic viruses and nanoparticles is usually discouraged as it removes the therapeutic from systemic circulation (except for the case of treating liver cancers) [32,33,34,35,36,37], there is a need for other methods of assessment. Measuring the activity of viable encapsulated Ad in the blood after circulation is a more direct method to evaluate in vivo protection against neutralizing antibodies as a model for the potential therapeutic efficacy of encapsulated and unencapsulated oncolytic Ad neutralizing environment. To directly measure the viable encapsulated or unencapsulated Ad in the blood, pre-immunized Balb/c mice were systemically administered Ad engineered with the GFP transgene, allowing measurement of GFP fluorescence in HEK293 cells transfected with the infected mouse serum and evaluate protection against neutralizing antibodies by measuring the transduction efficiency of each serum sample. A schematic of the entire protocol is shown in Supplementary Figure S1. Using Ad-GFP instead of Ad-β-gal, as undertaken by Yotnda et al. and Liu et al., for in vitro and in vivo neutralizing assays, allows for quicker assay time and removes the need to fix and stain cells and tissues [27,29]. By measuring the transduction efficiency of the isolated serum samples directly, the present study’s assay more accurately evaluates the protective capabilities of the liposome coating compared with liver transduction measurements in previous literature.

Liposome encapsulation is a viable method for protecting Ad from neutralizing antibodies when administering oncolytic Ad vectors systemically. Encapsulation of adenovirus was confirmed using cryo-TEM qualitatively, shown in Figure 7. Encapsulation rate was quantitatively measured at 96% by manually counting liposomes in cryo-TEM images, shown in Supplementary Figure S2 [24]. One possible mechanism of encapsulation is surface-tension-induced self-assembly, which suggests negatively charged adenoviruses are spontaneously encapsulated by small, empty cationic liposomes via self-assembly, thereby forming DfAd [24]. Hypothetically, the high surface tension of the small liposomes, and the charge interactions between the cationic liposome and the negatively charged adenovirus, drive the spontaneous encapsulation as shown in Supplementary Figure S3 [24,38]. Cryo-TEM images show partially encapsulated adenovirus, which suggest that viral particles can penetrate the lipid membrane to form DfAd [24]. Similar to the present study, previous studies using liposome encapsulation of Ad have demonstrated increased transduction efficiency in the presence of neutralizing serum in vitro when compared with unencapsulated Ad [27,28]. However, the present study reveals a novel correlation between liposome stability and neutralizing antibody protection by first quantifying the critical components of DfAd that affect protection against neutralizing antibodies of the encapsulating liposome. The optimization experiments in the present study demonstrate that DSPE-PEG2000-CA and cholesterol are critical components of DfAd that protect the Ad from neutralizing antibodies, as evidenced by significant reduction to the neutralizing antibody protection when removing either component from the DfAd formulation. Experiments also showed that PEG length correlates with neutralizing antibody protection, where DfAd with DSPE-succinyl acid provided the least protection against neutralizing antibodies while DfAd with DSPE-PEG10K-CA provided the most protection against neutralizing antibodies. To further investigate how these critical components affect DfAd, DLS, NTA, and Cryo-EM characterizations were used to measure the physical stability and morphology of DfAd when these components are removed.

Characterization of DfAd by DLS suggests that the nanoparticles become unstable at room temperature without DSPE-PEG2000-CA, manifested by significant increases in size and polydispersity index. Evidence from the NTA measurements without stirring shows that a similar observation is made between DfAd with and without DSPE-PEG2000-CA, where the size almost doubles without the PEG stabilizing the nanoparticles. Although cryo-EM images also show that the sizes of DfAd and DfAd without PEG do not change significantly, the sample without DSPE-PEG2000-CA appears more bunched together than DfAd with PEG. PEG is usually added to liposomes to increase stability and prevent flocculation and sedimentation, as it provides a physical barrier blocking other biomolecules and other liposomes from them [39,40,41]. Removing DSPE-PEG2000-CA from the formulation removes this barrier, causing them to bunch together, aggregate, and fuse, forming larger, more polydisperse nanoparticles, as observed from characterization experiments.

A similar aggregation effect is observed in DfAd when the PEG length is shortened significantly, as DLS showed by incorporating DSPE-PEG1000-CA, where the liposomes already start to grow and lose uniformity. This suggests that, by shortening the PEG chain below 2000 Daltons, the liposomes will destabilize and aggregate. Although this was not confirmed with NTA or cryo-EM, previous studies have shown that longer PEGs generally provide more physical stability for liposomes when compared with shorter PEGs, as they form a denser steric barrier from surrounding liposomes and biomolecules [42,43]. Lengthening PEG molecules on the surface of nanoparticles, however, is also shown to reduce their cellular uptake via the same steric hindrance mechanism that prevents nanoparticle agglomeration [44]. Therefore, PEG length selection is a balancing act between many different factors to improve delivery [45]. Therefore, although DSPE-PEG10K-CA demonstrated better protection against neutralizing antibodies compared with DSPE-PEG2K-CA, further studies are needed to determine whether PEG10K or PEG2K is better.

Figure 8B summarizes how PEG prevents aggregation between DfAd while removal results in aggregation between DfAd nanoparticles. The correlations between PEG concentration and in vitro antibody protection, as well as PEG length and in vitro antibody protection, bring to attention a potential direct link between physical stability and neutralizing antibody protection, one which requires further study. Previous studies have shown liposome aggregation and the membrane fusion that is due to poor stability can cause liposomes to become “leaky,” allowing encapsulated small molecule cargo to escape [46,47,48,49]. The “leaky” liposome theory could be a potential mechanism by which neutralizing antibodies bypass liposomes lacking PEG or grafted with short PEGs, motivating further investigation.

## 5. Conclusions

The present study demonstrates a novel method of evaluating neutralizing antibody protection by liposome-encapsulated Ad in immunized mouse models. By systemically injecting DfAd-GFP into preimmunized mice, extracting the blood, and transfecting HEK293 reporter cells with the infected serum, the in vivo neutralizing antibody protection assay assesses the protective capabilities of the DfAd-GFP liposome by measuring the fluorescence emitted from the transduced HEK293 cells. Based on the in vivo neutralizing antibody protection experiment results, DfAd can circulate in the blood without being neutralized for at least 5 min, allowing ~20 passes throughout the mouse body. The study also combined in vitro neutralizing antibody assays and characterization data to reveal a unique correlation between the structural stability of the liposome and neutralizing antibody protection by identifying DSPE-PEG and cholesterol as critical components for neutralizing antibody protection. The optimized DfAd is a viable platform to protect encapsulated Ad from neutralizing antibodies as it not only protects against neutralizing antibodies in diluted serum but protects against neutralizing antibodies in circulation in immunized mouse models. The results of this study provide novel methodologies and design insights for the clinical translation of systemic oncolytic viral therapy.

## Figures and Tables

**Table 1:** The effect of PEG Lipid composition and length on particle size using DLS of DfAd (*n* = 3).

Sample	z-Average (nm)	Fold Change (z-Average)	Polydispersity Index (PDI)	Fold Change (PI)
DfAd DSPE-PEG2K-CA	206 ± 2	1	0.1 ± 0.0	1
Df DSPE-PEG2K-CA (Empty Liposomes)	203 ± 2	1	0.1 ± 0.0	1
DfAd No DSPE-PEG-CA	3870 ± 57	19	0.7 ± 0.2	5
Df No DSPE-PEG-CA (Empty Liposomes)	3820 ± 175	19	0.4 ± 0.3	3
DfAd DSPE-PEG1K-CA	515 ± 27	3	0.6 ± 0.0	4
Df DSPE-PEG1K-CA (Empty Liposomes)	492 ± 12	2	0.6 ± 0.0	4
DfAd DSPE-PEG10K-CA	216 ± 1	1	0.2 ± 0.0	1
Df DSPE-PEG10K-CA (Empty Liposomes)	211 ± 2	1	0.2 ± 0.0	1

SEM is the standard error of the mean different *n* = 3 runs, not the distribution of the nanoparticle sizes.

**Table 2:** The effect of PEG Lipid composition and length on particle size of DfAd using NTA (Without Stirring).

Sample	Mean Diameter (nm)	Fold Change (Diameter)	D10, D50, D90 (nm)
DfAd DSPE-PEG2K-CA	136	1.0	73, 120, 215
DfAd No DSPE-PEG-CA	291	2.1	127, 230. 560

**Table 3:** The effect of PEG Lipid composition and length on particle size of DfAd using NTA (With Stirring).

Sample	Mean Diameter (nm)	Fold Change (Diameter)	D10, D50, D90 (nm)
DfAd DSPE-PEG2K-CA	145	1.0	82, 125, 234
DfAd No DSPE-PEG-CA	134	0.9	79, 121, 202
DfAd DSPE-PEG1K-CA	128	0.9	68, 105, 223
DfAd DSPE-PEG5K-CA	165	1.1	90, 148, 264

## Data Availability

The data presented in this study are available in articles and Supplementary Materials.

## Supplementary Materials

The following supporting information can be downloaded at https://www.mdpi.com/article/10.3390/pharmaceutics17060769/s1, Figure S1: Schematic of the in vivo neutralizing antibody protection assay; Figure S2: Encapsulation efficiency of DfAd-GFP; Figure S3: Schematic of liposome encapsulation of adenovirus; Figure S4: Additional characterization and structural analysis of DfAd.
