# Supplementary material for: Adenoviruses Encapsulated in PEGylated DOTAP-Folate Liposomes Are Protected from the Pre-Existing Humoral Immune Response
Source: Pharmaceutics. 2025 Jun 11;17(6):769. doi: 10.3390/pharmaceutics17060769 (PMC12196153; doi:10.3390/pharmaceutics17060769)
Supplement: Supplementary file 1 [file pharmaceutics-17-00769-s001.zip › Supplementary Figure S1.pdf]

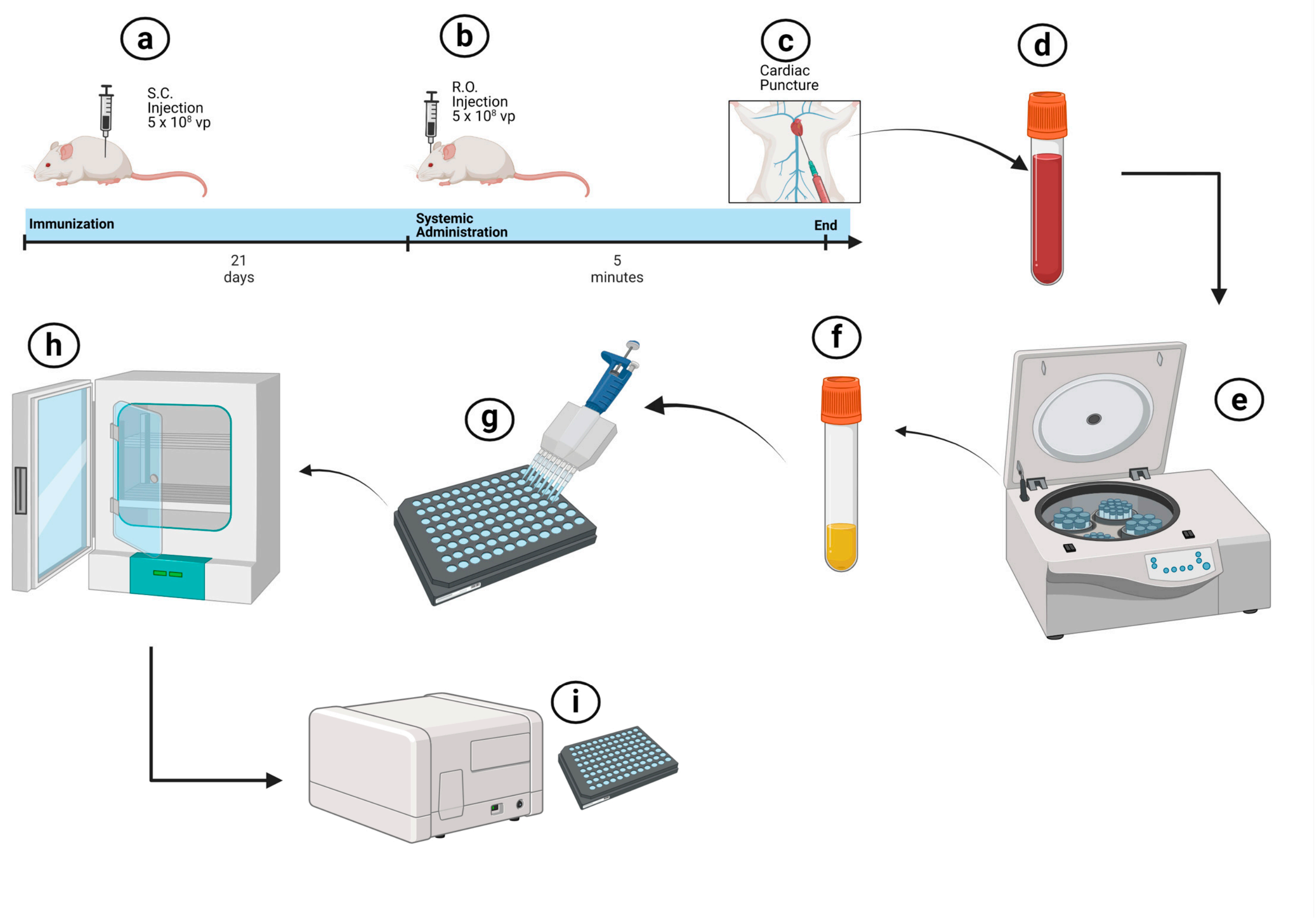

Supplementary Figure S1. Schematic of the In Vivo Neutralizing Antibody Protection Assay. Figure shows an overview of the different steps of the in vivo neutralizing antibody assay described in Figure 4. (a) Balb/c mice are immunized for 21 days with one subcutaneous injection of Ad or PBS. (b) Mice are injected retro-orbitally with Ad-GFP or DfAd-GFP, which are allowed to circulate for five minutes. (c) Blood is extracted from mice by cardiac puncture and transferred to (d) heparin blood collection tubes. (e) Blood is transferred to Eppendorf tubes, incubated on ice for 30 minutes, and centrifuged at 3,000 RPM for 10 minutes. (f) The resulting serum is isolated into separate tubes on ice. (g) HEK293 cells pre-plated 24 hours earlier are treated with the resulting serum and (h) incubated at 37°C for 24-48 hours. (i) The resulting fluorescence was measured using a fluorescence plate reader. Created in BioRender. Phung, A. (2025) https://BioRender.com/k19q694
