# Supplementary material for: Adenoviruses Encapsulated in PEGylated DOTAP-Folate Liposomes Are Protected from the Pre-Existing Humoral Immune Response
Source: Pharmaceutics. 2025 Jun 11;17(6):769. doi: 10.3390/pharmaceutics17060769 (PMC12196153; doi:10.3390/pharmaceutics17060769)
Supplement: Supplementary file 1 [file pharmaceutics-17-00769-s001.zip › Supplementary Figure S2.pdf]

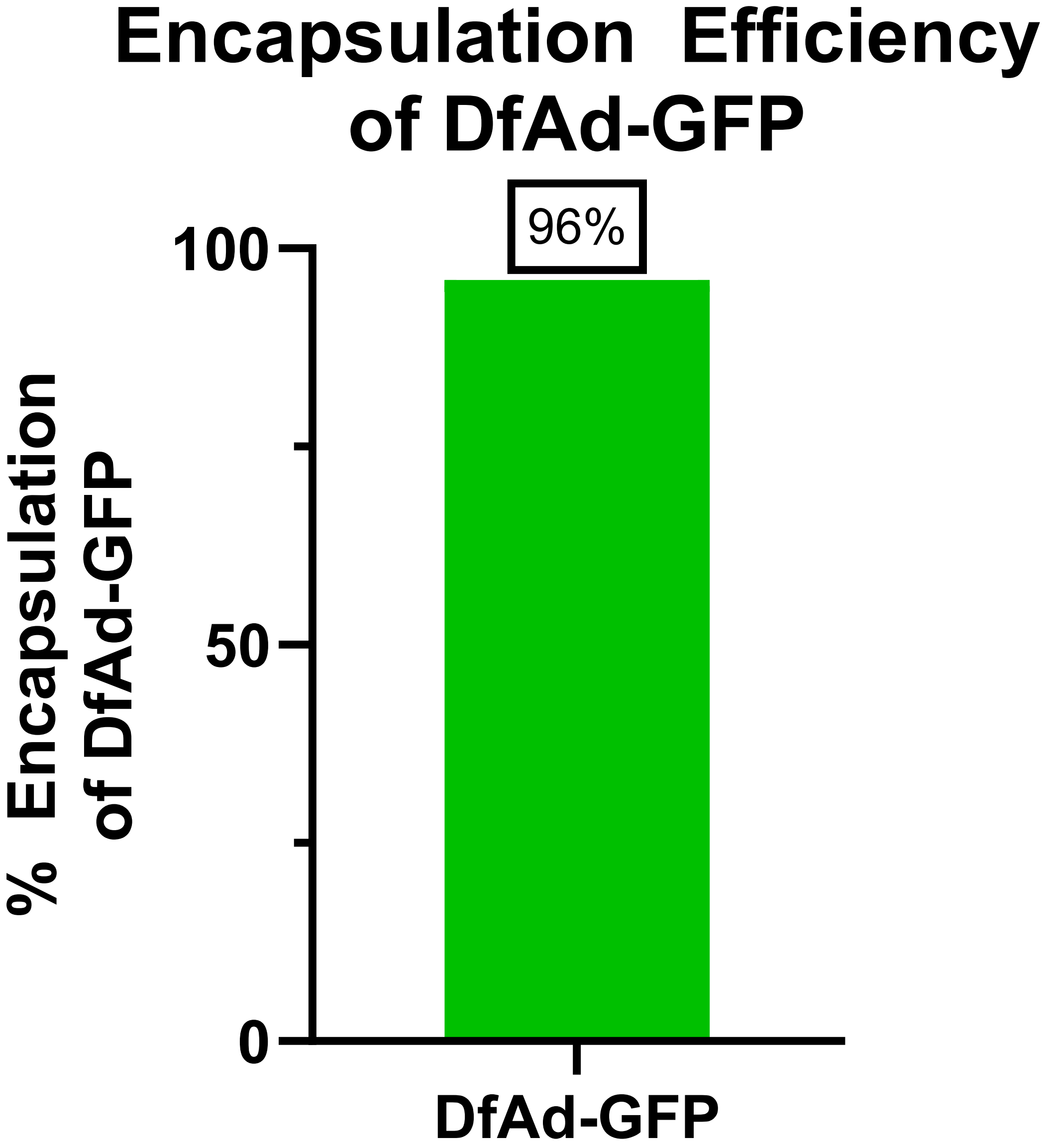

Supplementary Figure S2. Encapsulation efficiency of DfAd-GFP. The encapsulation efficiency of DfAd-GFP was measured at 96% by manually counting cryo-EM micrographs.
