# Supplementary material for: Adenoviruses Encapsulated in PEGylated DOTAP-Folate Liposomes Are Protected from the Pre-Existing Humoral Immune Response
Source: Pharmaceutics. 2025 Jun 11;17(6):769. doi: 10.3390/pharmaceutics17060769 (PMC12196153; doi:10.3390/pharmaceutics17060769)
Supplement: Supplementary file 1 [file pharmaceutics-17-00769-s001.zip › Supplementary Figure S3.pdf]

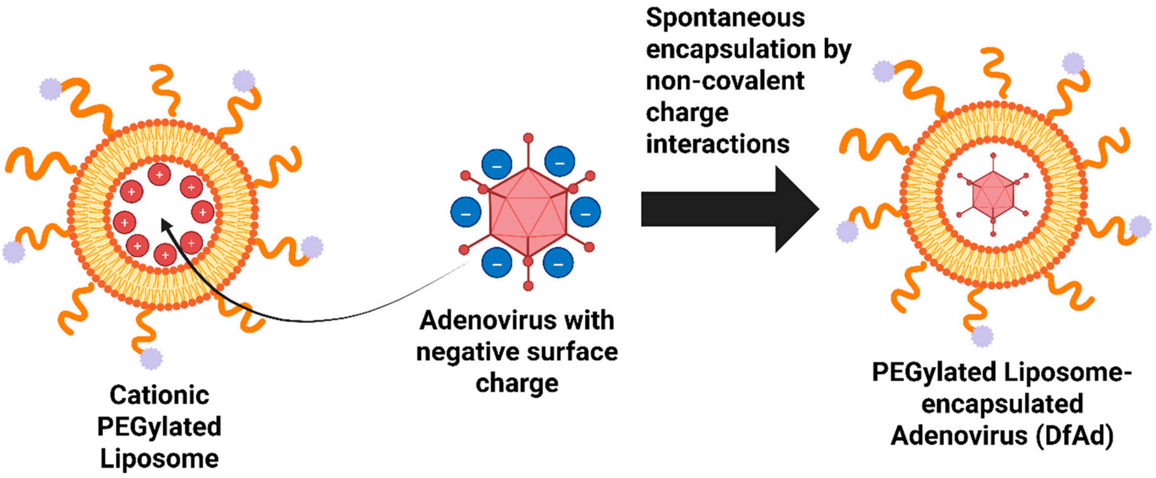

Supplementary Figure S3. Schematic of liposome encapsulation of adenovirus. The spontaneous encapsulation of adenovirus by PEGylated liposomes is hypothetically occurs due to favorable charge interactions between the negatively-charged hexon proteins of the adenovirus and the positively-charged liposome. Created in BioRender. Phung, A. (2025) https://BioRender.com/a1gghgc
