# Supplementary material for: Adenoviruses Encapsulated in PEGylated DOTAP-Folate Liposomes Are Protected from the Pre-Existing Humoral Immune Response
Source: Pharmaceutics. 2025 Jun 11;17(6):769. doi: 10.3390/pharmaceutics17060769 (PMC12196153; doi:10.3390/pharmaceutics17060769)
Supplement: Supplementary file 1 [file pharmaceutics-17-00769-s001.zip › Supplementary Figure S4.pdf]

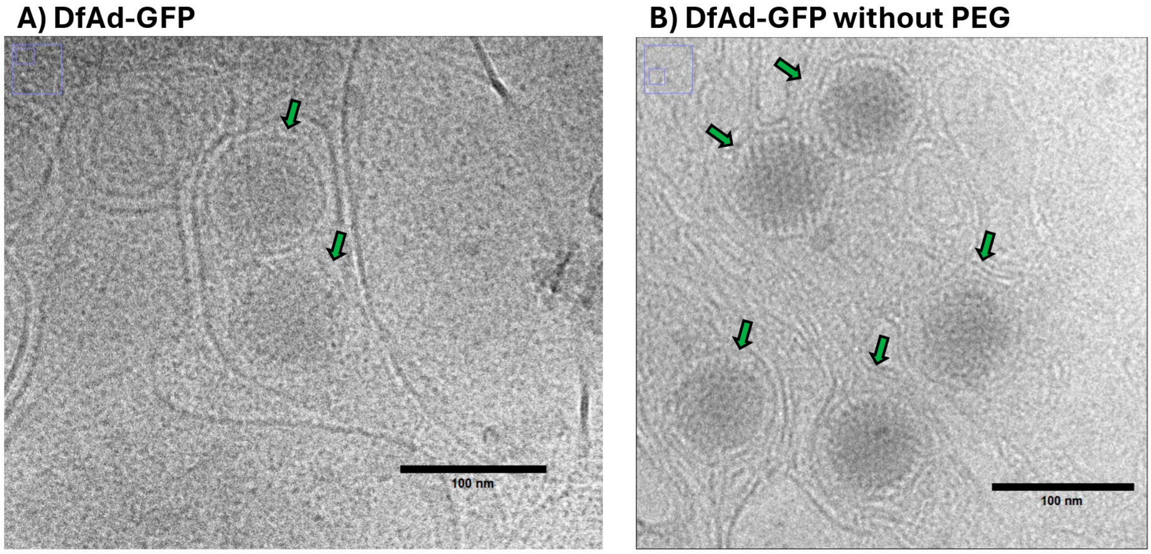

Supplementary Figure S4. Additional Characterization and Structural Analysis of DfAd. (A) Cryo-TEM image of DfAd at 30,000× magnification plus a 50% digital zoom. Green arrows point to liposome-encapsulated Ad-GFP. This image appears to show two separate liposome-encapsulated Ad-GFP that are encapsulated together in a liposome. (B) Cryo-TEM image of DfAd-GFP without PEG at 30,000× magnification plus a 50% digital zoom. Green arrows point to liposome-encapsulated Ad-GFP. This image demonstrates tightly-enveloped DfAd without PPEG in a less bunched up group. In all figures, the scale bar for Cryo-TEM images is 100 nm.
